# Nanoparticle role on the repeatability of stimuli-responsive nanocomposites

**DOI:** 10.1038/srep06624

**Published:** 2014-10-15

**Authors:** Sungsook Ahn, Sang Joon Lee

**Affiliations:** 1 Biofluid and Biomimic Research Center, Pohang University of Science and Technology, Pohang, 790-784, Korea; 2 Department of Mechanical Engineering, Pohang University of Science and Technology, Pohang, 790-784, Korea

## Abstract

Repeatability of the responsiveness with time is one important concern for effective durable functions of stimuli-responsive materials. Although the increase in the yield and tensile strength of the hybrid composite materials by nanoparticle (NP) incorporation has been reported, exact NP effect on stimuli-responsiveness is rarely reported. In this study, a set of nanoscale actuating system is demonstrated by a thermo-sensitive process operated by polyethylene glycol (PEG) linked by gold nanoparticle (AuNP). This designed nanocomposite exclusively provides an artificial on/off gate function for selective passages of permeate molecules. The results demonstrate high repetition efficiency with sharp responding in a timely manner. In terms of the morphology changes induced by repeated swelling-deswelling mechanics, the nanocomposite exhibits phase separation between AuNP clusters and PEG domains. This leads to a delay in responsiveness in a cumulative way with time. Acting as stable junction points in the nanocomposite network structures, the incorporated AuNPs contribute to maintain repeatability in responsiveness. This study contributes to new-concept smart material design and fundamental understanding on the hybrid nanomaterials for various applications in terms of a dynamic mechanical behavior.

Smart or stimuli-responsiveness^1^ that modifies properties in response to external stimuli represents a growing cadre of materials that support various applications, including controlled-release agents^2^,^3^, responsive coatings^4^, and adaptive shape-memory materials^5^. In addition to the separation of molecules by size and charge for which traditional membranes are employed, responsive membranes such as human skin demonstrate a smart valve function controlled by external stimuli^6^. Recent advances in nanotechnology have been interested in designing smart materials that mimic the processes found in natural systems. In this point, synthetic membranes capable of responsive functions find broad ranges of applications.

Different from homogeneous polymeric materials, nanocomposites composed of metal nanoparticles (NPs) dispersed in a polymer matrix exhibit novel properties^7^. Composed into a responsive composite material, the role of each component is cooperative leading to characteristic collective property. In this point, blends and chemically-linked particle-polymer systems are physically different in many aspects. Composite materials are widely reported to upgrade stiffness and strength of a polymeric materials without a significant loss in resilience or toughness. One example, adding mica to nylon produces a five-fold increase in the yield and tensile strength of the material^8^,^9^. Even though precise control of NP dispersion in the polymeric matrix has been focused, this is typically hurdled by thermodynamic instability and easy agglomeration as a result of high surface free energy of NPs. Recent advances exploit both the enthalpic and entropic interactions that direct the spatial distribution of NPs in a polymeric matrix to enhance macroscopic performance of materials. Even though versatile noncovalent methods have been employed to assemble individual NPs into designed structures^10^,^11^,^12^, NPs have been covalently collected for increased stability and uniformity. Once covalently linked, NP-embedded nanocomposite generates unique physical properties contributed by collective interactions of individual NPs^13^.

The responsive mechanism of stimuli-responsive materials depends on many factors such as conformational changes and phase segregation of the employed polymers as well as polymer–water/polymer–polymer interaction in the practical temperature ranges^13^,^14^,^15^,^16^,^17^,^18^,^19^. In terms of responsiveness, the properties of the composite materials are complicated due to compositional complexity. Recent advances exploit both enthalpic and entropic interactions to direct the spatial distribution of NPs and thereby control the macroscopic performance of the material. NPs influence the phase-separation kinetics and significantly alter the coarsening dynamics of polymer mixtures^20^. Theoretical studies of NP-filled mixtures suggest the existence of distinct pattern formation at early stages of phase separation^21^,^22^ and a subsequent slowing of domain growth at later times^23^. Experiments have confirmed a substantial slowing of phase separation by NP addition to a polymer mixture^24^,^25^. In this study, the role of NPs in a stimuli-responsiveness of nanocomposites is investigated in terms of stable repetition of the mechanistic swelling-deswelling mechanistic cycles. The role of NPs in the composite materials are investigated in terms of the dynamical structural change and durability in mechanical responsiveness.

## Results

**Figure 1:**
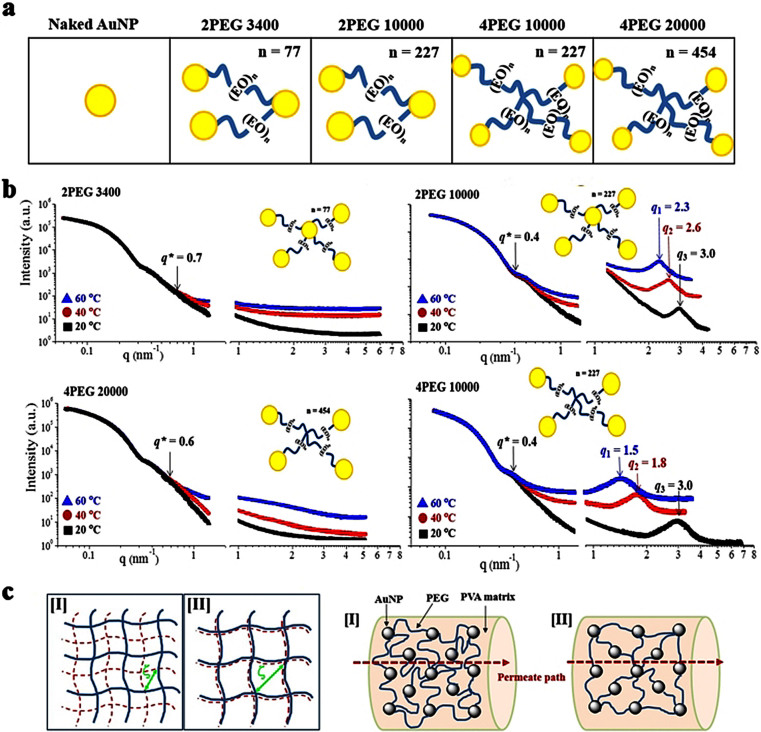
(a) Gold nanoparticle (AuNP) and structure of the functional polyethylene glycol (PEG) for interlinking. Due to multi reactive sites of the AuNP to thiol group of PEGs various networked structures are generated. Binary functional PEGs having number of EO unit as of n = 77 is 2PEG 3400 and n = 227 is 2PEG 10000. Quaternary functional PEGs having EO unit as of n = 227 is 4PEG 10000 and n = 454 is 4PEG 20000. (b) Small angle X-ray scattering (SAXS) results of the designed AuNP-PEG nanocomposites in solution state in broad *q* ranges (two SDD distance conditions are combined) for 2PEG 3400, 2PEG 10000, 4PEG 10000 and 4PEG 20000 linked AuNP clusters. There is a critical *q* value (marked by q*) from which temperature-responsiveness is diversified indicating dual regions of the designed network: stable large scale domain and responsive small scale domain. In two systems of 2PEG 10000 and 4PEG 10000, definable size is determined according to the temperature. (c) Illustration of the dual-responsive network structures. Bold lines denote stable connection, while dotted lines indicates flexibly responsive chains by the external stimuli (left). Pore size variation of the nanocomposites embedded in PVA matrix induced by the stimuli-responsive PEGs through which permeates are transported. Swollen PEGs generates smaller path for permeate molecules [I], while shrunken PEGs allow relatively wide pathway for effective transport of permeates [II].

Citrate-stabilized colloidal AuNP (naked AuNP) of 20 nm average diameter are prepared^26^,^27^ in aqueous standard solution adjusted to around 2.4 × 10^12^ AuNPs/mL (Supporting Information). Binary and quaternary thiol end-capped functional polyethylene glycol (PEG) are incorporated for AuNP interconnection (Figure 1a)^10^,^11^. Molecular weight between the junction point (M_p_) is controlled by their characteristic structures: 2PEG 3400 has M_p_ = 3400 ((EO)_n_, n = 77) and 2PEG 10000 has M_p_ = 10000 ((EO)_n_, n = 227) for binary-functional PEGs. Meanwhile, 4PEG 10000 has M_p_ = 10000 ((EO)_n_, n = 227) and 4PEG 20000 has M_p_ = 20000 ((EO)_n_, n = 454) for quaternary-functional PEGs. In addition, the number of incorporated PEG molecules is controlled to ×10, ×50, and ×100 times of the AuNPs for each system, considering multi-reactive sites on a single AuNP (Supporting Information). Network is hardly formed if binary PEG monomers are only activated. However, interconnected composite clusters are formed as a result of multiple reactivity of AuNP surface to thiol end groups of the PEGs. NPs are typically considered to perform a random walk on the lattice model (Figure S1)^28^. Effective particle mobility on the lattice is controlled by specific particle-to-solvent step ratios^29^. Considering that each lattice contains one AuNP, and these lattices are diversely interconnected according to the molecular weight and the structure of the PEGs. Multiple PEG linkages are attached to the AuNP surface to form networked assemblies. Mono-tethered AuNPs are analogous to single-tailed surfactants or diblock copolymers, whereas multi-tethered AuNPs work as junction points in the network. They also possess additional levels of complexity and anisotropy that can be exploited in self-assembly. The crosslink density (ρ) of the fully-linked network is inversely proportional to the molecular weight between the junction points (M_p_).

Figure 1b presents small-angle X-ray scattering (SAXS) results conducted at the 4C beam line of the Pohang Accelerator Laboratory (Pohang, Korea) with the designed AuNP−PEG nanocomposite networks in aqueous solution. Aqueous PEG solutions exhibit unique physical property depending on the molecular weight: the solubility decrease and/or phase separation occurs above the critical temperature (lower critical solution temperature, LCST)^2^,^3^,^30^,^31^. Different from typical responsive materials exhibiting isotropic changes in their scales, the organic−inorganic hybrid composites display deviation from the typical rubber elasticity by characteristic contribution of individual components. The nanocomposites designed in this study display dual regions for responsiveness: a large-size domain is fixed while a small-scale domain is responsive to an environmental stimuli. At a fixed PEG concentration (×100), temperature-responsiveness of PEGs are compared at three different temperature conditions (20, 40 and 60 °C) (Figure 1b). For all the systems there is a characteristic *q* value (q*) from which the spectrum is diverged responding to each temperature condition: q* = 0.7 nm^−1^, 0.4 nm^−1^, 0.4 nm^−1^ and 0.6 nm^−1^ for 2PEG 3400, 2PEG 10000, and 4PEG 10000 and 4PEG 20000, respectively. From those q*, the solubility of the composite network decreases according to the temperature increase from 20 to 60 °C, exhibiting characteristic LCST, except lowest molecular weight 2PEG 3400 system^30^,^31^. This indicates that the designed AuNP−PEG composites consist of dual domains: non-responsive stable domain in large-scale (low *q* region) and stimuli-responsive flexible domain in small-scale (high *q* region). However, size-definable structures are generated only for 2PEG 10000 and 4PEG 10000 system marked at *q*_1_, *q*_2_, and *q*_3_, for each temperature condition. This moves to lower *q* region with temperature increase, exhibiting larger size formation at increased temperature. With the increase in the incorporated PEG amount, the q* point moves to the higher *q* region, indicating smaller size scale, from which the materials become responsive (Figure S2). We suggest this dual regions come from the characteristic hardness of the embedded metal nanoparticle and also simultaneously responsive polymeric matrix, and this combined properties are unique in composite materials.

We obtained same batch of the nanocomposites and after purification we obtained characteristic physical properties (SAXS, UV-vis, TEM, XNI, XMI) of the designed nanocomposites and used them for all the mass transport experiments. Temperature-responsive nano-scale networks are illustrated in Figure 1c. The designed nanocomposites are embedded in poly(vinyl alcohol) (PVA) gel matrix (3 wt%, M_w_ ~ 72,000 Merck), keeping the temperature-responsiveness of nanocomposites without leakage and shape deformation, significant swelling or shrinking in the designed experimental conditions^32^. Even at increased temperature several times above and below the transition point, AuNP−PEG composites released from the PVA matrix are not detected. The AuNP−PEG nanocomposite embedded in PVA matrix exhibits differentiated paths for the selected permeates according to the temperature condition. Structural transition occurs by triggered temperature depending on the nanocomposite structures. The correlation length (ξ), the distance between the junction points of the network, changes according to the temperature: compared with the condition of lower temperature of highly swollen state [I], the ξ values become larger by the shrinkage at an increased temperature [II]. According to the temperature change, the structure of the designed nanocomposite is effectively controlled for the transport of permeate molecules. With increased temperature, the selected permeates pass the nanocomposites more effectively through larger ξ [II] than through smaller ξ at lower temperature [I].

**Figure 2:**
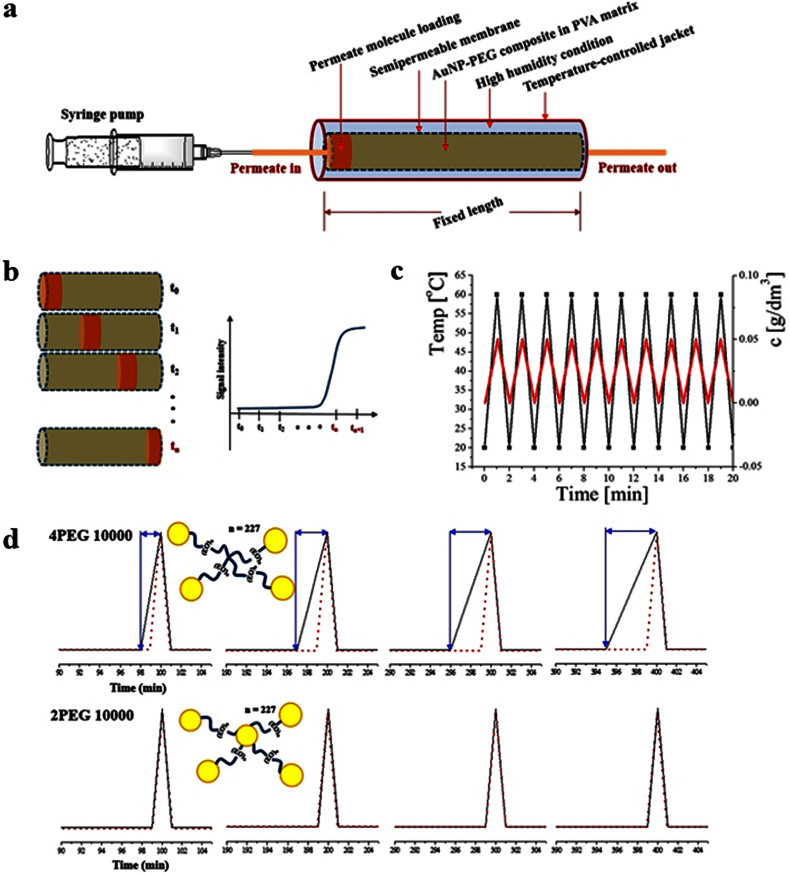
(a) Experimental set-up for mass-transport. Permeate molecules are loaded at the entrance of the diffusion cell and flow rate are carefully controlled. (b) Retention time (t_n_) of each system is determined from which the permeate molecules are detected at the outlet flow. (c) Repetition cycles of the temperature triggering to the nanocomposite-loaded diffusion cell and molecular responding of permeate detection. The results are obtained by Rhodamine 6G passing through 2PEG 10000 nanocomposites and recorded in a minute interval. (d) Time-dependent repetition cycles for two nanocomposite systems of 2PEG 10000 and 4PEG 10000. Temperature triggering and permeate detection occur simultaneously for 2PEG 1000 system, while there is cumulative delay for 4PEG 10000 system. All the figures were created by the authors.

Mass transport is controlled in a designed diffusion cell in which selected permeate molecules (Rhodamine 6G or Pyrene)^33^,^34^ pass through the designed nanocomposite. The AuNP–PEG nanocomposite embedded in PVA matrix is loaded in the humidity- and temperature-controlled glass tube of 0.1 cm diameter and 5 cm length and connected to the syringe pump (Figure 2a). The retention time (t_n_) of the permeates is recorded by direct observation of the eluted solution color or fluorescence signal in continuous batch (Supporting Information). Relatively sharp point of t_n_ is determined by the time from which the presence of the permeate at a detectable concentration is verified (Figure 2b). For all the designed systems, there is a sharp increase in the signal determined as a retention time (t_n_). To determine t_n_ for each nanocomposite switching on/off temperature-sensitive valve, nanocomposite packing geometry and flow rate are carefully controlled at two temperature conditions (20 and 60°C) switched in a minute interval. The controlled low rate of 0.001 μL/min through 2PEG 10000 nanocomposite, proper repetition signals of Rhodamine 6G are obtained in a timely manner as illustrated in Figure 2c. The temperature changes are recorded in a minute interval marked by solid black lines, and sharp increase in the signals of permeate molecules are exclusively detected during the condition of an elevated temperature.

The synchronized signals are displayed in Figure 2d, reflecting the repeatability of the temperature-responding valve function of the designed nanocomposites. The on/off switching is controlled regularly by detected permeate molecules of a fixed concentration. The temperature change is recorded with a minute interval (black line) and the detected t_n_ of permeate molecule (dotted red line) is controlled accordingly. The time-dependent swelling-deswelling cycling repetition is compared for 2PEG 10000 and 4PEG 10000 systems. At an initial stage, the repetition cycle is regular for both systems. 2PEG 10000 system shows unchanged repetition pattern during the measurement time duration. However, 4PEG 10000 system exhibits delayed responsiveness between the temperature onset and permeate elution response as time goes on, which is quantified by the length of the arrows.

**Figure 3:**
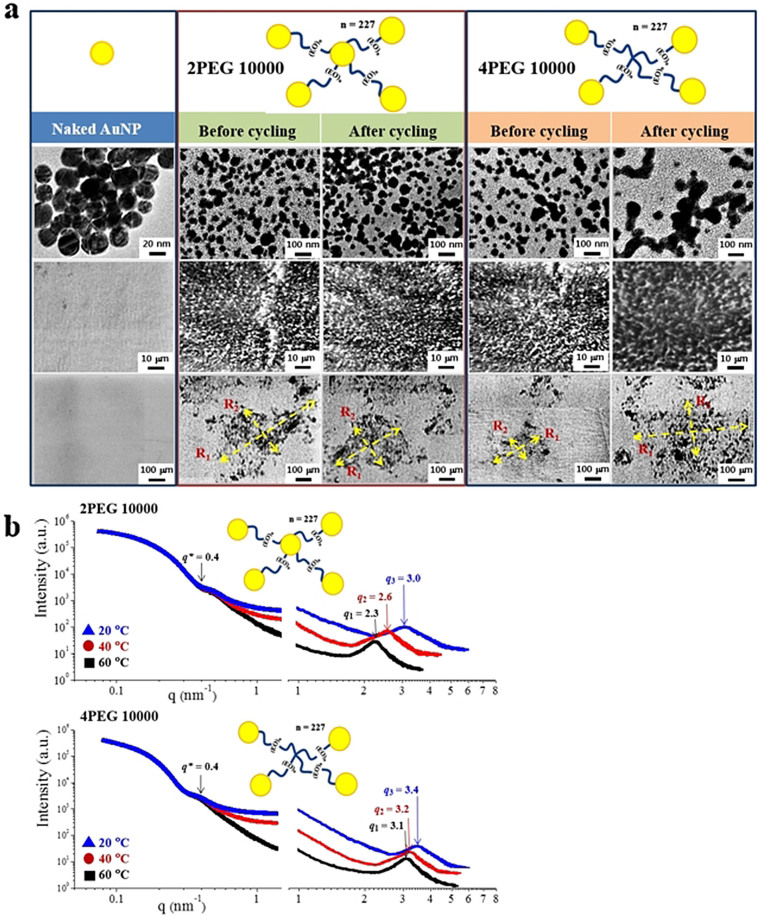
(a) TEM (top line), XNI (middle line) and XMI (bottom line) images before and after the repetition cycling for 2PEG 10000 and 4PEG 10000 system. (b) SAXS results after the repetition cycling for 2PEG 10000 and 4PEG 10000 systems.

The structures of the employed AuNP–2PEG 10000 and AuNP–4PEG 10000 clusters are compared before and after the cycling repetitions (Figure 3a). The measurement temperature is controlled as 20 °C. At the first line of the transmission electron microscopy (TEM) images, the AuNPs in the composite clusters are effectively contrasted by high electron density against the linked organic PEGs. A representative image of the citrate-covered spherical AuNPs without ligand linkage (naked AuNPs) verifies 20 nm average diameter of a single AuNP. Before the cycling repetition, the AuNPs connected by binary 2PEG 10000 show rather more closely packed clusters compared with those connected by quaternary 4PEG 10000 of longer interparticle distances. Even after the cycling of continuous valve work until around 500 min, the AuNP clusters connected by 2PEG 10000 have no clear difference. Meanwhile, 4PEG 10000 exhibits prominently distinguishable changes which become highly aggregated AuNP clusters in TEM images. The structural difference in nanoscale composite cluster is dominated by M_p_ as well as the structure of the linker-molecules. By the quaternary structure, 4PEG 10000 actually has M_P_ value of 20000 crossed at the center, this induces polymer-dominated phase behavior against the AuNP-linked hybrid structures.

In the second line of Figure 3a, X-ray nano imaging (XNI) results are displayed. The images are obtained at the 7C beam line in PAL (Supporting Information). Beam size is adjusted in 100 μm × 100 μm at 7 keV energy. Spatial resolution is approximately 200 nm and field-of-view (FOV) is adjusted to 20 μm × 20 μm (the images are put together to get a whole image). Before cycling, both type of AuNP clusters exhibit homogeneous small dots. After the repetition cycling, 2PEG 10000 linked system does not generate prominent difference. Meanwhile, dotted patterns of 4PEG 10000 linked clusters become prominently larger after the cycled repetition by induced phase separation of AuNPs from the PEG polymer domain.

In the third line of Figure 3a, the X-ray micro imaging (XMI) results obtained at the 6D beam line at PAL provide a larger FOV than XNI (Supporting Information). Spatial resolution is approximately 2 μm and FOV is adjusted to 600 μm × 600 μm. Au displays high contrast in X-ray images by its high X-ray absorption coefficient^27^. At this imaging condition discrete unit clusters are detected, by which the size of a single cluster can be evaluated. Increased M_p_ of the PEG enhances cluster size with broad interconnection. As the inset arrows in the images illustrate, the average cluster size is evaluated using the arithmetic mean of horizontal (R_1_) and vertical (R_2_) cluster diameters. Assuming that the cluster is an ellipsoid, the longer length R_1_ is determined on a cluster and then the shorter R_2_ is made at the center point of the R_1_ at a perpendicular angle. Cluster sizes averaged by ten clusters for each case are summarized according to the PEG type and the concentration (Figure S1c). In terms of the cycling repetition effect, 2PEG 10000 system does not generate prominent change. However, 4PEG 10000 system exhibits larger cluster in size after the cycling.

Figure 3b shows the SAXS results of the 2PEG 10000 and 4PEG 10000 systems after the cycled repetition until around 1000 min. There is no big difference in the peak position of the 2PEG 10000 system before (Figure 1a) and after the cycled repetition (Figure 3b). However, the peaks of the 4PEG 10000 move to the higher *q* region, indicating structural shrinkage as a results of the cycled repetition. In addition, the size difference induced by the temperature becomes smaller, reflecting temperature insensitivity. These structural changes strongly affect the delayed response to the temperature change in switching function. This SAXS result confirms that the ξ shrinks through the mechanical repetition of responsive cycling, induced by the locally concentrated PEG domains in the nanocomposite. The solubility of PEGs in aqueous condition is a function of the temperature, molecular weight, concentration, additives and so on^35^. For the designed nanocomposites, the temperature-responsiveness is dominantly affected by the tailored M_p_ between the junction points. However, the local concentration of the PEGs in the nanocomposite network is changed by the structural dynamics. Through repeated swelling-deswelling mechanics, the structure of the nanocomposite is rearranged into lower energy state where there is an inherent phase separation of the metallic AuNP regions from the polymeric PEG regions. With induced phase separation, the temperature-responsiveness of the nanocomposite is delayed in a cumulative way (Figure 2d).

**Figure 4:**
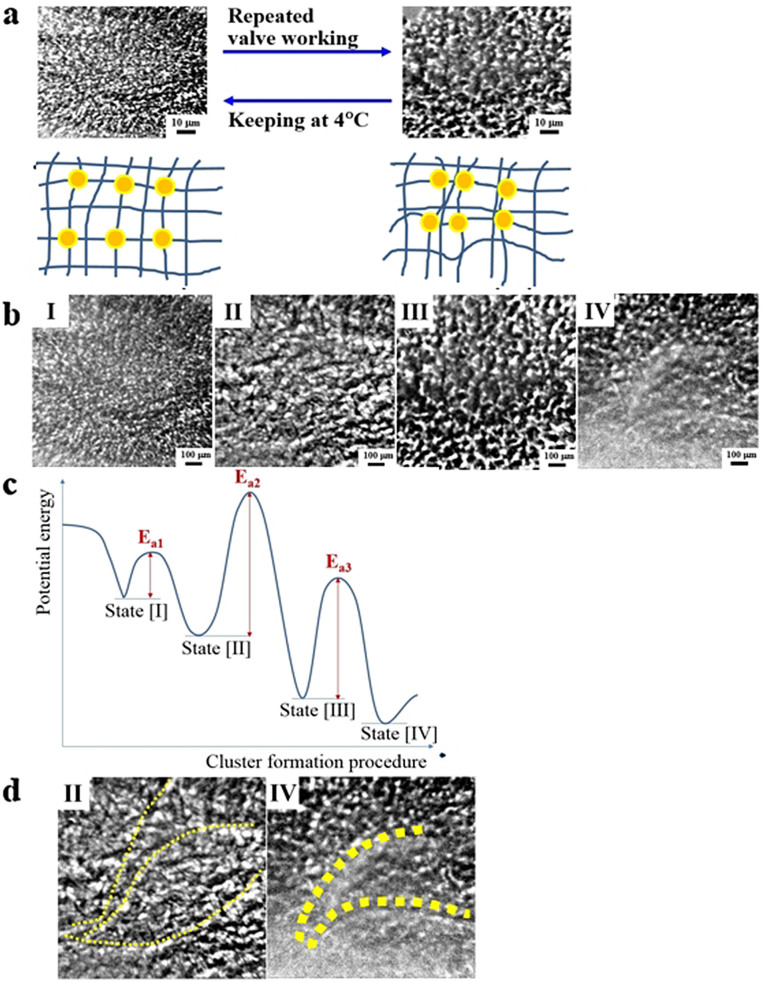
(a) XMI image and schematic illustration of the nanocomposite before (left) and after (right) the cyclic repetition. This is a reversible procedure controlled by temperature control. (b) XMI images of nanocomposites obtained with mechanistic repetition from [I] to [IV]. (c) Suggested energy level and activation of the state [I] to [IV] in (b). Due to high activation energy, the state [III] and [IV] is reversible. (d) The wrinkled lines are drawn on the image [III] and [IV].

Once the AuNP and PEG are chemically linked together, the structure of the nanocomposite is determined even though physical rearrangement occurs further by responsive mechanics. To confirm the time delay in responsiveness is caused by the physical rearrangement of cluster structures, the structure is reorganized by dispersing AuNPs in the nanocomposite homogeneously. The temperature-insensitive 4PEG 10000 nanocomposites are kept at 4 °C for a week (with a sonication once a day) and then the AuNPs are redispersed in the nanocomposite modulated by the increased PEG solubility at this low temperature condition. The structural changes are provided in Figure 4a by the XMI images and the corresponding schematic network illustrations. The aggregation−dispersion of AuNPs is a reversible procedure and this determines temperature-responsiveness of the nanocomposite. The designed nanocomposite structures are dominated by the controlled PEG solubility, in which chemically embedded AuNPs are reorganized reversibly. However, keeping the nanocomposite at high temperature (60°C) for more than a week does not induce the phase separated state shown in Figure 4a at the right side. Therefore, it is only by the repeated mechanical processes that modifies the clustering behaviors of chemically linked NP arrangements in the polymer network. Those mechanistic repetitions cannot be reversible simply by temperature-induced solubility control of the polymer.

## Discussion

Inherent properties of responsive materials are induced either by solubility transition or conformation of a macromolecule under certain conditions modulated by external stimuli^31^. The polymer chains in composite materials can be stretched and aligned. This molecular mobility is a typical feature of soft or responsive materials. Typical shape modulation known as rubber deformation is uniaxial based on conventional rubber theory. Length increases in an isotropic way based on λ = *L*/*L*_0_ (where *L*_0_ is the initial length and *L* is the length after stretching), thus width and thickness decrease by the relation of λ^1/2^. Scale changes in conventional responsive materials are also considered isotropic based on rubber elasticity. However, changes in composite materials deviate from this isotropic deformation due to compositional complexity. Partial responsiveness of the nanocomposites designed in this study maintains shapes in large-scale while activates valve function in small-scale. This is highly beneficial to be employed for selective mass transport control without physical deformation in macroscale but with effective smart valve performance in nanoscale. The temperature-responsiveness is inherently induced by polymeric domain of PEGs. However through the repeated mechanical cycling of the responsiveness, the polymer domain becomes more concentrated by phase separation where the metallic AuNP domain and polymeric PEG domain are distinctively identified. This induced structural change causes time delay in a cumulative way. With further prolonged mechanistic repetition, the structure of the nanocomposite obtained by XMI changes in a sequence displayed in Figure 4b. Starting with state [I], more aggregated larger cluster of state [II] and [III] with further time. The nanocomposite reaches the state [IV] where there is a more prominent phase separation between the AuNP cluster region and polymeric film region. The state [III] and [IV] are physically irreversible, thus they do not return to the state [I] by any temperature-controlled aging steps.

Temperature-responsive PEG induces controlled valve functions in the nanocomposite, simultaneously generates phase separation by repeated mechanistic procedures. The nanocomposites having proper nodes composed of the AuNP junctions maintain their structures thus the repeated mechanistic functions. Therefore, properly repositionable NPs chemically embedded in nanocomposites are suggested to be at a metastable state [I] (Figure 4c). Phase-separated state [II] is at the lower energy level. It can be reversibly reached by temperature control due to relatively low activation energy (E_a1_) to be easily overcome. However, even though the aggregated sates [III] and [IV] are positioned at the lower energy state, they cannot be reached by physical control by temperature increase due to their relatively high activation energy (E_a2_ and E_a3_). To overcome this energy barrier, mechanistic swelling-deswelling repetition performed in this study is suggested. Due to inherent incompatibility between the NPs and PEGs, the phase separated state is most stable even though they are chemically linked. Therefore, in the limit of possible condition, the AuNPs are aggregated while PEGs from film-like polymeric aggregation. On the images of the state [III] and [IV], phase separated regions are marked by the inset lines demonstrating characteristic wrinkle formation after the repeated mechanistic swelling-deswelling procedures (Figure 4d). The wrinkled lines become bolder with more cycling repetition.

In summary, smart nanocomposites are designed which exhibit high selectivity in mass transport in nanoscale and sharp temperature-responding in a designed time. With structural rearrangements, the nanocomposites display delayed response by repeated mechanical swelling-shrinking procedures. These procedures are similar to those occurring in natural systems where there is a lifetime in life functions. One of the most prominent characteristics of the living systems is a lifetime. A tiredness limits the life function and eventually causes to death at a certain time. Before complete stop (death), the cycling of the live function typically becomes retarded. The designed responsive materials showing characteristic lifetime implies these natural behaviors similarly occurring in biological systems. The designed system in this study is different from the NP-polymer blend system where confinement induces enhancement of NP dispersion^36^. Therefore, the designed system in this study deals with the stimuli-responsiveness rather than thermodynamics dominant for NP phase-separation from polymer domain. Until a certain state, the procedures are reversible but the structural changes and the responding patterns become irreversible with higher deformation in nanoscale structures. To keep the timely-working stimuli-responsiveness, the AuNPs working as stable junction points in the nanocomposite networks are important. This contribution of NP junction points in the stimuli-responsive materials are exclusively reported in this study. The detailed relations between the responding time delays and structural changes are further to be investigated. This study newly illuminates dynamic aspects of the stimuli-responsive materials for broad potential applications.

## Methods

### Small-angle X-ray scattering (SAXS)

Synchrotron SAXS measurements are performed at 4C beam line of PAL equipped with a position-sensitive two-dimensional (2D) detector. Two energy setting are employed for wavelength modulation: 10 keV (0.0675 nm^−1^) and 18 KeV (0.1217 nm^−1^). Samples of 1 mm thick are used by stacking the five 200 μm-thick Si wafer of SiN_3_ sample window. The sample-to-detector distance (SDD) as of 4 m covers the *q* range of 0.0679 nm^−1^ < *q* < 1.64094 nm^−1^, where *q* = (4π/λ)sin(θ/2) is the magnitude of the scattering vector and θ is the scattering angle. The *q* range is calibrated using polystyrene-*block*-poly(ethylene-*ran*-butylene)-*block*-polystyrene (SEBS) (*q* = 0.19165 nm^−1^). On the other hand, the 1 m SDD covers the *q* range of 0.346 nm^−1^ < *q* < 7.68039 nm^−1^. The *q* range is calibrated using silver behenate (*q* = 1.052 nm^−1^). A W/B4C double multilayer monochromator are installed to deliver monochromatic X-rays with 6.75 nm (18360 keV) wavelength and spread of Δλ/λ = 0.01. The 2D scattered X-rays are recorded by a CCD camera (Mar CCD, Mar USA, Inc., CCD165). The collected SAXS data are corrected by subtracting the data of background and empty cell scattering. For the system design durability and safety of the system are importantly considered to get reasonable repeatability of the designed nanocomposite. Too high temperature is not aimed not to induce water evaporation of the designed nanocomposite system. Under the given experimental conditions of three ambient temperature condition of 20, 40 and 60°C, we fortunately get noticeable SAXS peak diversification.

### Synchrotron X-ray nanoscopy (XN)

X-ray imaging is useful for *in situ* observation of samples. Since vacuum application which is applied in typical image method can distort or further damage AuNP clusters. In this study because of its unique filed-of-view, reasonable resolution and safety for the samples, X-ray imaging is actively employed. Experiments were carried out at the 7C beam line of the 3rd generation synchrotron radiation source of the PAL. The X-ray source of 10^11^ photons/μm^2^/sec consists of undulator with 20 mm period and 70 poles. The beam size is 100 μm × 100 μm at 7 keV. The X-ray source is radiated from a 3 GeV bending magnet and then monochromatized using a Ge(111) DCM. For focused images, monochromatic X-ray beam of nominally selected 7 keV is focused on the sample using a condenser zone-plate (CZP, 1 mm dia. Beryllium refractive compound lenses) with innermost and outermost diameters of 4 nm and 100 nm, respectively. The primary X-ray image is magnified 50 times using an objective zone plate lens (140 μm innermost and 50 nm outermost diameter, W) and is converted into a visible image on a thin scintillator crystal (Tb:LSO, 20 μm thickness). The visible image is further magnified × 20, using an optical microscope, providing a total magnification of ×1000 image on a cooled CCD camera (Princeton Instrument VersArray 1300B cooled CCD) of 1340 pixel × 1300 pixel, which generates an equivalent field of view of 21 μm × 21 μm.

### X-ray microscopy (XM)

Synchrotron X-ray images were captured at 6D beam line of the PAL. The X-ray source is a bending magnet with critical energy of 8.7 keV at 3 GeV electron energy operation. The white beam is attenuated by polished beryllium (Be) of 0.5 mm thickness or polished Si wafer of 1 mm thickness. The primary X-ray image is converted into a visible image on a thin scintillator crystal CdWO4 of 100 μm thickness. X-ray images were captured using a CCD camera (PCO, PCO2000). The field-of-view with a 10× objective lens in front of the camera was approximately 3.6 mm × 2.4 mm in physical dimension. The pixel size is about 0.9 μm.

## Author Contributions

S.A., S.J.L. developed the concepts. S.A. designed and perform the experiments. S.A. analyzed the results and wrote the paper. All the authors confirm the final version of the manuscript.

## Supplementary Material

Supplementary Information Supporting Information
