# Supplementary material for: Nanoparticle role on the repeatability of stimuli-responsive nanocomposites
Source: Sci Rep. 2014 Oct 15;4:6624. doi: 10.1038/srep06624 (PMC4197417; doi:10.1038/srep06624)

# Nanoparticle role on the repeatability of stimuli-responsive nanocomposites

Sungsook Ahn1,2 and Sang Joon Lee1,2,*

1Biofluid and Biomimic Research Center, 2Department of Mechanical Engineering Pohang University of Science and Technology, Pohang, 790-784, Korea.

*Correspondence

Tel: +82-54-279-2169, Fax: +82-54-279- 3199, E-ma[il: sjlee@postech.ac.kr](mailto:sjlee@postech.ac.kr)

# Supporting Information

**Preparation of spherical AuNPs of 20 nm in average diameter.** To prepare gold nanoparticles (AuNPs), gold chloride (III) trihydrate (HAuCl4⋅3H2O) is dissolved in DI Milli- Q water (1.0 × 10-3 mol/L) under refluxing. Sodium citrate tribasic dihydrate solution in DI water (4 × 10-2 mol/L) is added to the above solution. Reaction completion is detected by color changes from pale yellow to wine red. Boiling condition is further maintained for 15 min after the color change is completed and then cooled to room temperature. The AuNP solution is dialyzed overnight using Spectra/Por®7 membrane (1,000 Da cut) against DI Milli-Q water to remove excess sodium citrate tribasic dihydrate. The AuNP solutions are placed on a typical copper grid and then dried under air at room temperature for transmission electron microscopy (TEM) (JEOL Cs-corrected HR-TEM, JEM-2200FS). Given that the organic layers of the AuNPs are not clearly detected by the electron beam in TEM, gold cores with high electron density in each AuNPs are captured distinctively. From this TEM image, the average diameter of the formed AuNPs is adjusted to be approximately 20 nm.

**Preparation of interconnected AuNPs by PEGs.** Thiol end-capped polyethylene glycol (PEG) ligands are added at the second step. Bi-functional and four-armed PEGs are purchased (Laysan Bio, Arab, AL, USA) and used without further purification. After the size of the AuNP is determined by the aforementioned method, designed amount of ligand aqueous solution is added to the above aqueous solution to modify the surface properties of the citrate-covered AuNP and stirred at room temperature or higher (between 50 °C and 60

°C) for 6 h to 12 h until no further color change is observed. The un-reacted residue ligands are minimized to be less than 1 ppm by testing aliquot of the samples. The AuNP solutions are dialyzed overnight by Spectra/Por®7 membrane (25K Da cut) against Milli-Q water for purification. The aqueous surface-modified AuNP stock solutions are adjusted to have a concentration of about 2.4 × 1018 AuNPs/m3 in consideration of the diameter of AuNPs (average 20 nm in diameter) and the HAuCl4 concentration of 1.0 × 10-3 mol/L. Given that the standard concentration of the AuNP stock solution of 1.0 mmol/L, the concentration of the ligand stock solution is controlled to 10 mmol, 50 mmol and 100 mmol (Figure S2).

The physical property of the AuNP clusters formed by PEG inter-linking is investigated by UV-vis spectrum. Physically hybrid NP clusters typically red-shift (such as natural aging). The UV-vis spectra of designed AuNPs tethered by a constant PEG chain length 10000 but in different linear-, binary-, and quaternary structures are compared (**Figure S1a**). The AuNPs linked by linear PEG generate surface plasmon at approximately λ = 540 nm, which is similar to that of the single AuNP of 20 nm diameter. However, the AuNPs linked by binary and quaternary PEGs shift to λ = 610 nm and λ = 630 nm, respectively. The UV-vis spectrum of the designed PEG-AuNP nanocomposite clusters closely resembles that of physical NP aggregation. In addition, the 4PEG-linked clusters red-shift more effectively than 2PEG- linked ones. Characteristic light-responsiveness of AuNP clusters changes according to the structure of the interlinking molecules.

NPs are typically considered to perform a random walk on the lattice model. In addition, effective particle mobility on the lattice is controlled by specific particle-to-solvent step ratios. The lattice model illustrated in **Figure S1b** is adopted to describe the inter-linked AuNP−PEG composite clusters. Each lattice contains one AuNP, and these lattices are diversely interconnected according to the molecular weight and structure of the PEGs.

Multiple PEG linkages are attached to the surface of the AuNPs to form assemblies. Mono- tethered AuNPs are analogous to single-tailed surfactants or diblock copolymers, whereas multi-tethered AuNPs work as junction points in the polymer network. They also possess additional levels of complexity and anisotropy that can be exploited in self-assembly. The crosslink density (ρ) of the fully-interlinked network is inversely proportional to the Mw between junction points (Mp).

**Figure S1. (a)** UV-vis spectroscopy of the AuNPs of 20 nm in diameter linked by PEGs of different structure but the same arm length of 10000. The AuNPs linked by a linear PEG 10000 exhibits UV-vis absorbance at 540 nm, while those linked by binary and quaternary 2PEG 10000 and 4PEG 10000 have far longer wavelength of 610 and 630 nm, respectively.

**(b)** Lattice model to describe the structures of the AuNP-PEG nanocomposite networks. Each AuNP locates in a lattice and PEGs interconnect those AuNPs. **(c)** Variation of the cluster size of the AuNPs linked by 2PEG 3400, 2PEG 10000, 4PEG 10000 and 4PEG 20000 with the concentration of ×10, ×50, ×100 (number of PEGs/number of AuNPs). The cluster size is averaged from 100 clusters of the XNI images. Numbers on the graph means PEG concentration for each case. The standard deviation is marked as light blue bar for each case.

## a b c


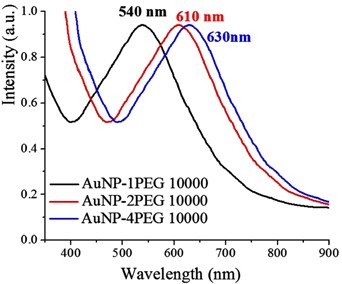

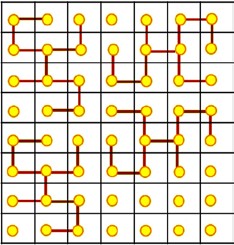

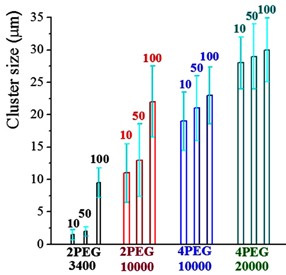


**Figure S2.** AuNP and PEG ratio effect obtained by SAXS. The aqueous surface-modified AuNP stock solutions are adjusted to have a concentration of about 2.4 × 1018 AuNPs/m3. Given that the standard concentration of the AuNP stock solution of 1.0 mmol/L, the concentration of the PEG ligand stock solution is controlled to 10 mmol (×10), 50 mmol (×50) and 100 mmol (×100). **(a)** 2PEG 3400 **(b)** 2PEG 10000, **(c)** 4PEG 10000 and **(d)** 4PEG

20000. Beyond the critical *q** value the systems are responsive to the temperature. However, below that *q** value all the systems are stable. The *q** values move to the higher *q* region with increased PEG amount for all systems, indicating smaller size region from which responding to the external stimuli.

## a b


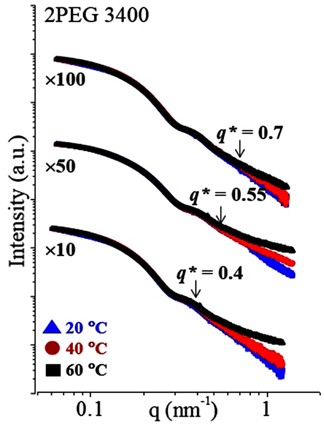

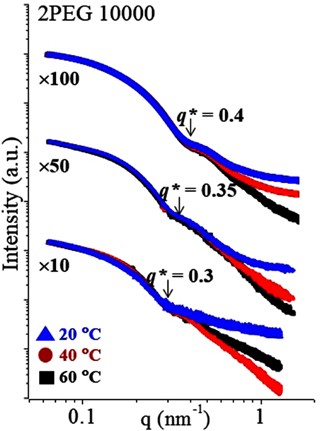


## c d


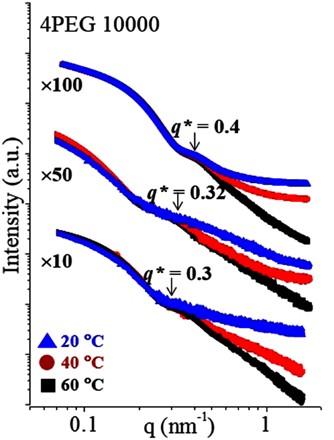

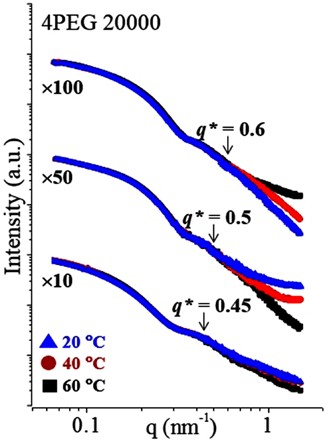


**Small-angle X-ray scattering (SAXS).** SAXS measurements are performed at the 4C synchrotron SAXS beam line of PAL. Samples of 1 mm thickness are used by stacking five Si wafer sample holders having 200 μm-thick SiN3 window at the center. The sample-to- detector distance is fixed at 3 m, covering the *q* range of 0.04 nm-1 < *q* < 0.75 nm-1, where *q*

*=* (4π/λ)sin(θ/2) is the magnitude of the scattering vector and θ is the scattering angle. The *q* range is calibrated using SEBS [polystyrene-*block*-poly(ethylene-*ran*-butylene)-*block*- polystyrene]. The collected SAXS data are corrected by subtracting the data of background and empty cell scattering. All measurements are performed at the isothermal condition of 20 °C. The designated *q* range is focused on the polymeric networked structures of PEGs, not on the size of the AuNP. Therefore, the *q* region displayed in this study does not overlap with those of the AuNPs (if there is no specific mention); hence, the peak comes purely from the polymeric network cluster structures. The results of the higher *q* range mentioned in this study which corresponds to the AuNPs, are not presented. Instead, the size of the AuNPs is discussed based on the TEM results.


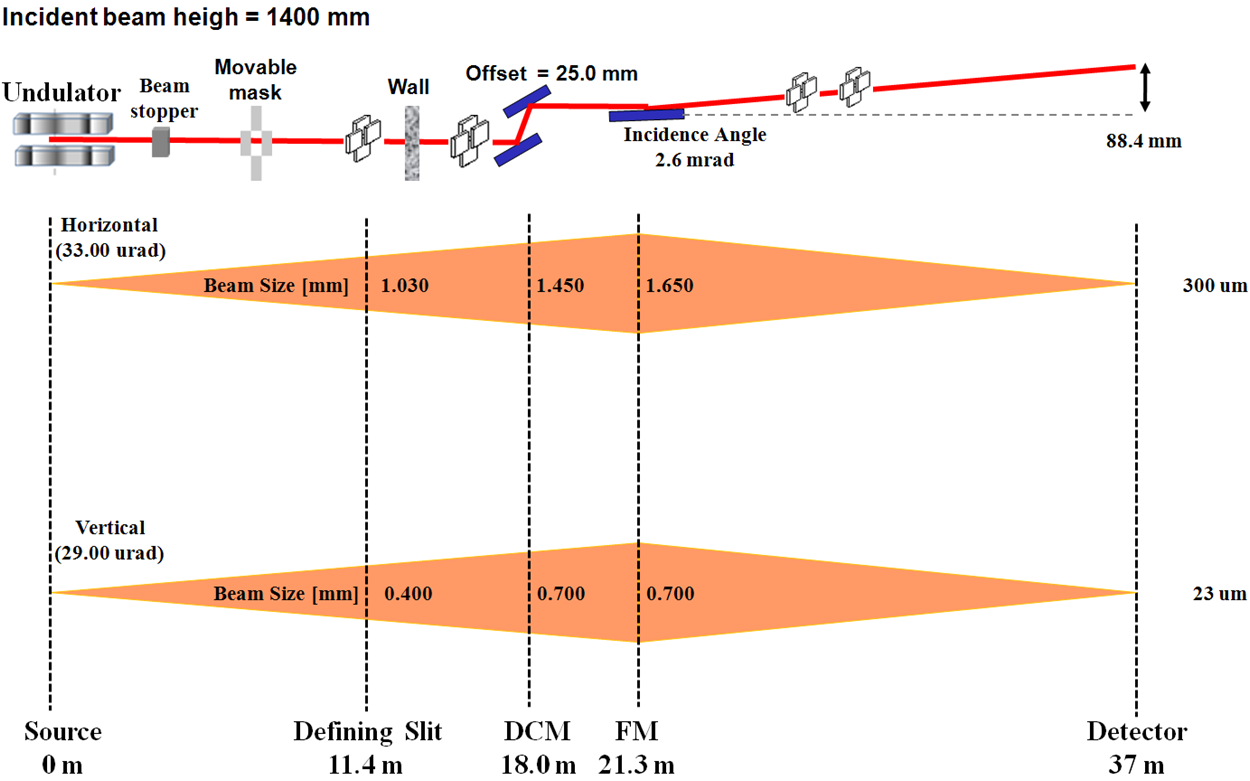


**Figure S3.** Experimental set-up for small angle X-ray scattering (SAXS) at PAL

**Synchrotron X-ray nanoscopy (XN)***.* Experiments are carried out at the 7C beam line of PAL. The X-ray source of 1011 (photons/μm2/sec) is consisted of an undulator with 20 mm period and 70 poles. The beam size is 100 μm × 100 μm at 7 keV. The X-ray beam source is radiated from a 3 GeV bending magnet and then monochromatized using a Ge(111) DCM. For focused images, monochromatic X-ray beam of nominally selected 7 keV is focused on the sample using a condenser zone-plate (CZP, Beryllium refractive compound lenses of 1 mm diameter) with innermost and outermost diameters of 4 nm and 100 nm, respectively. A stainless steel annular ring aperture (AA) with 2.4 mm inner and 3.2 mm outer diameters is used to obtain a hollow-cone type X-ray illuminates on a sample necessary for the Zernike phase-contrast imaging. An order sorting aperture made of a tungsten plate is used not only to pass the focused beam but also to block the 0th and higher order diffracted X-rays. An imaging zone-plate is fabricated with 1.6 µm-thick gold rings on a silicon nitride (Si3N4) membrane of 100 nm thickness. The depth of focus of the objective zone plate is 200 µm at 7 keV, for which the practical sample thickness is limited to less than 200 µm. For the phase- contrast imaging, a gold plated ring-shaped phase plate (PP) array is located at the downstream of the objective lens, and the ring dimensions (114.6 µm) are matched with that of the AA located at the CZP. For an experimental set-up of large filed-of-view, CZP is removed to get the diverged beam on the sample. The thickness of the PP is about 0.7 µm, which corresponds to the λ/4 phase delay for positive phase contrast. The primary X-ray image is magnified 50 times using an objective zone plate lens (140 μm innermost and 50 nm outermost diameter, W) and is converted into a visible image on a thin scintillator crystal (Tb:LSO, 20 μm thickness). The visible image is further magnified × 20, using an optical microscope, providing a total magnification of ×1000 image on a cooled CCD camera (Princeton Instrument VersArray 1300B cooled CCD) of 1340 pixel × 1300 pixel resolution, which corresponds to an equivalent field of view of 21 µm × 21 µm in physical size. Silicon (Si) wafer holder has silicon nitride (SiN3) membrane window at the center is used as a sample holder. The figure is created by the authors.


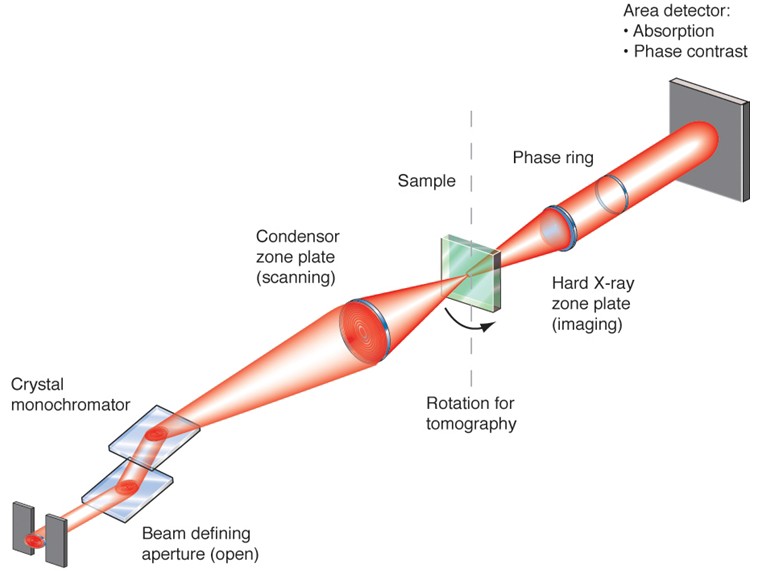


**Figure S4.** Experimental set-up for X-ray nanoscopy (XN) at PAL

**Two-dimensional (2D) X-ray microscopy (XM).** Synchrotron X-ray images are captured at 6D beam line of the Pohang Accelerator Laboratory (Pohang, Korea). The X-ray source was a bending magnet with a critical energy of 8.7 keV at 3GeV electron energy operation. The white beam was attenuated by a polished beryllium (Be) of 0.5 mm thickness or polished Si wafer of 1 mm thickness. The photon energy (E) was about 22 keV with energy resolution (ΔE/E) of 15.8 keV to 34.4 keV (~ 84 %). The brightness was 22 × 1012 (ph/s/mm2) and the beam size was about 30 mm (H) × 5 mm (V). The samples were placed at approximately 30 m downstream of the source, whereas the detector was placed downstream of the objects at 30 cm. The size of the X-ray beam illuminating the test sample was adjusted to that of the field-of-view using a slit module to avoid unnecessary exposure of X-ray beam on the sample. An attenuator made of aluminum (Al) sheets was located at the beam inlet of the experiment hutch on the way of X-ray propagation pathway to attenuate light intensity, even when no image was photographed. This attenuation of light intensity protected the sample and detector from strong X-ray irradiation. The primary X-ray image was converted into a visible image on a thin scintillator crystal CdWO4 of 100 μm thickness. X-ray images were captured using a CCD camera (PCO, PCO2000). The field-of-view with a 10× objective lens attached in front of the camera was approximately 3.6 mm × 2.4 mm in physical dimension. The pixel size was about 0.9 μm.


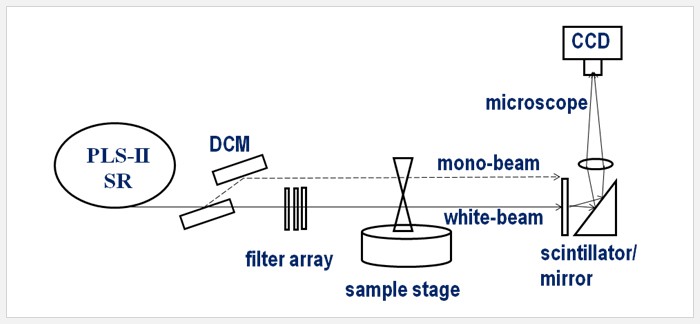


**Figure S5.** Experimental set-up for X-ray microscopy (XM) at PAL.

**Pyrene or Rhodamine transport through AuNP-PEG composites in aqueous solution.** Rhodamine 6G is an ionic molecule of high water solubility exhibiting dark orange color in water and often used as a tracer to track the rate and direction of flow in water. On the other hand, Pyrene is a nonionic hydrophobic molecules and is sensitive to the fluorescence detection. A stock solution of Pyrene (water solubility: 0.135 mg/L) is prepared by adding a known weight of the compound in 20 wt% ethanol in water. The mixture is sonicated to yield a clear solution. The experimental 2 μM solution of Pyrene is prepared from it by dilution wherein the ethanol concentration is 0.5%. Such a small concentration of the ethanol is considered not to affect the stimuli-responsiveness of AuNP-PEG nanocomposites. With high water solubility (400 g/L), Rhodamine 6G is directly dissolved in water and used as a saturated solution. AuNP-PEG composites solutions are loaded in a Spectra/Por7 membrane bag (1000 Da cut) and put in a temperature-controlled tube as shown in Fig. 4a. The volume and the height of the AuNP-PEG composite embedded PVA matrix (Mw~72,000 Merck, 3 wt %) is carefully designed. Pyrene dissolved solution 3 mL is load on top of the tower and then aliquot samples are collected at every minute. A series of collected samples are measured by fluorescence spectra which are recorded using a spectrophotometer. A Perkin Elmer Luminescence Spectrometer L550B System with FL Winlab 4.0 software is used to obtain all fluorescence excitation and emission spectra. Solutions are held in Agilent Technologies Open-top UV Quartz Cells (10 mm by 10 mm) with 3.0 mL of volume. Spectra are obtained with a scan rate of 100 nm/s and intensities are collected at 0.5 nm intervals. An emission wavelength of 428 nm, an excitation wavelength of 326 nm, and slit widths of 2.50 nm for both emission and excitation are used for Pyrene spectra. Pyrene shows significant absorbance at this wavelength hence its use as an excitation wavelength. For Rhodamine 6G spectra, an excitation wavelength of 485 nm, an excitation slit width of 10 nm, and an emission slit width of 2.50 nm are used. All spectra are obtained at 25 °C. In terms of the tn of Pyrene and Rhodamine, hydrophobic Pyrene shows longer tn compared with that of hydrophilic Rhodamine showing effective water tracking.

**Figure S6**. **(a)** Cluster size (I and II) and pore size in a cluster (III and IV) are considered. **(b)** Cluster size vs. retention time (tn), **(c)** Temperature vs. tn and **(d)** 1/*q* vs tn effect on the transport of two dyes (Pyrene and Rhodamine 6G) through each designed PEG−linked AuNP clusters. The applied flow is controlled by gravity.

## a


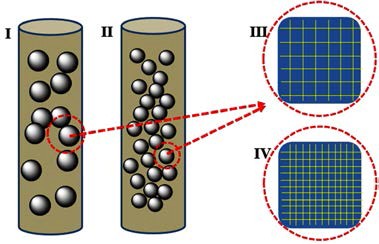


**b**


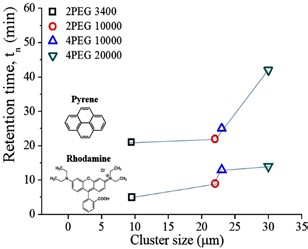


**c**


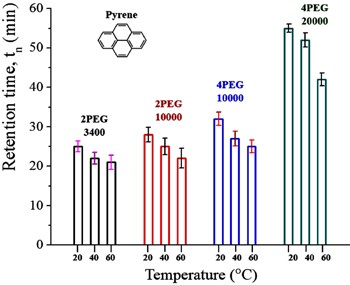

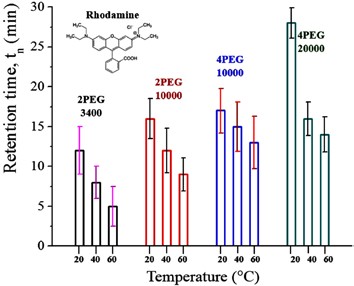


## d


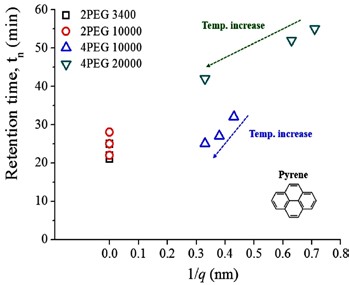

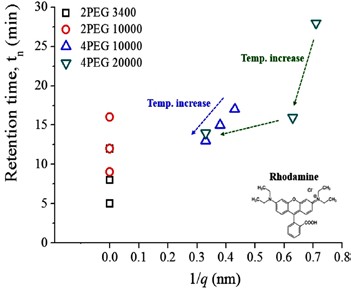

Supplement: Supplementary Information — Supporting Information [file srep06624-s1.docx]
